# Antiarrhythmic calcium channel blocker verapamil inhibits trek currents in sympathetic neurons

**DOI:** 10.3389/fphar.2022.997188

**Published:** 2022-09-15

**Authors:** S. Herrera-Pérez, L. Rueda-Ruzafa, A. Campos-Ríos, D. Fernández-Fernández, J.A. Lamas

**Affiliations:** ^1^ Laboratory of Neuroscience, CINBIO, University of Vigo, Vigo, Spain; ^2^ Grupo de Neurofisiología Experimental y Circuitos Neuronales, Hospital Nacional de Parapléjicos, SESCAM, Toledo, Spain; ^3^ Laboratory of Neuroscience, Galicia Sur Health Research Institute (IISGS), Vigo, Spain

**Keywords:** verapamil, TREK, superior cervical ganglion, riluzole, TREK-2

## Abstract

**Background and Purpose:** Verapamil, a drug widely used in certain cardiac pathologies, exert its therapeutic effect mainly through the blockade of cardiac L-type calcium channels. However, we also know that both voltage-dependent and certain potassium channels are blocked by verapamil. Because sympathetic neurons of the superior cervical ganglion (SCG) are known to express a good variety of potassium currents, and to finely tune cardiac activity, we speculated that the effect of verapamil on these SCG potassium channels could explain part of the therapeutic action of this drug. To address this question, we decided to study, the effects of verapamil on three different potassium currents observed in SCG neurons: delayed rectifier, A-type and TREK (a subfamily of K2P channels) currents. We also investigated the effect of verapamil on the electrical behavior of sympathetic SCG neurons.

**Experimental Approach:** We employed the Patch-Clamp technique to mouse SCG neurons in culture.

**Key Results:** We found that verapamil depolarizes of the resting membrane potential of SCG neurons. Moreover, we demonstrated that this drug also inhibits A-type potassium currents. Finally, and most importantly, we revealed that the current driven through TREK channels is also inhibited in the presence of verapamil.

**Conclusion and Implications:** We have shown that verapamil causes a clear alteration of excitability in sympathetic nerve cells. This fact undoubtedly leads to an alteration of the sympathetic-parasympathetic balance which may affect cardiac function. Therefore, we propose that these possible peripheral alterations in the autonomic system should be taken into consideration in the prescription of this drug.

## Introduction

Some drugs used in the treatment of heart diseases, like verapamil or nifedipine, have been developed and are used based on their interaction with the L-type voltage-gated calcium channel, expressed by cardiac and vascular smooth muscle cells. This is the case for class IV antiarrhythmic drugs used in pathological conditions such as chronic angina pectoris, cardiac arrhythmias or hypertension (Kato et al., 2004). In fact, the phenylalkylamine verapamil exerts its therapeutic action predominantly on cardiac cells, reducing heart contractility and rate (Cohen et al., 1987), by blocking L-type calcium channels (Keith et al., 1994; Bergson et al., 2011). Notwithstanding, verapamil has also been shown to exert a significant inhibition of T-type calcium channels expressed in both native and heterologous systems (Freeze et al., 2006; Bergson et al., 2011). Although attracting less attention, those calcium antagonists also inhibit several potassium channels. Concentrations in the low micromolar range inhibit delayed rectifier (*I*_KDR_; K_V_1.3, K_V_1.5) and ether a go-go (*I*_Keag_; K_V_11.1) potassium currents in both native and heterologous systems (Chouabe et al., 1998; Rauer and Grissmer, 1999; Baba et al., 2015; Diesch and Grissmer, 2017).

The effect of verapamil and analog drugs on potassium channels has also been verified in native heart cells. In fact, verapamil inhibits the acetylcholine-induced potassium current (*I*_KAch_) in atrial myocytes (Ito et al., 1989). However, and despite the importance that the voltage sensitive A-type potassium current (*I*_A_) has both in the heart (Liu et al., 2011; Devenyi et al., 2017; Grandi et al., 2017) and in the autonomic nervous system (ANS) (Lamas et al., 1997; Doan and Kunze, 1999), the potential effect of verapamil modulating these channels has not been investigated. TREK-1, TREK-2 and TRAAK constitute the TREK subfamily of K2P family. These channels (mainly TREK-2) are abundantly expressed in the superior cervical ganglion (SCG) neurons (Cadaveira-Mosquera et al., 2011) and also in atrial and ventricular cardiomyocytes (Limberg et al., 2011; Bond et al., 2014; Bodnar et al., 2015), where they function as major regulators of the resting membrane potential (RMP) (Cadaveira-Mosquera et al., 2011; Unudurthi et al., 2016; Grandi et al., 2017). Indeed, TREK channels have been related to various heart diseases (Schmidt et al., 2012; Wiedmann et al., 2016; Decher et al., 2017) and although it has been previously shown that verapamil blocks some members of the K2P family, including TRESK (Park et al., 2018) and TASK-4 (Staudacher et al., 2018), its effect on TREK channels remains unknown.

It is common to check the effect of substances that affect the functioning of the heart on non-neuronal cardiac cells, mainly cardiomyocytes and comparatively, less effort has been devoted to test the interaction of drugs with the neurons regulating heart working. However, we know that the neurons of the parasympathetic intracardiac ganglion (ICG) and sympathetic SCG innervate both muscle fibers and pacemakers. The SCG hosts a critical population of sympathetic neurons projecting to the cardiac tissue (Pather et al., 2003; De Gama et al., 2012), and several studies have suggested that the SCG might be directly implicated in the pathophysiology of different cardiovascular diseases (Hernandez-Ochoa et al., 2009; Kong et al., 2013; Na et al., 2014; Cheng et al., 2018).

In the present study, we have used cultured sympathetic neurons, isolated from the mouse SCG (mSCG), to study the effects that verapamil produces on their RMP and excitability. Using the Patch-Clamp technique we found that verapamil evokes a dose-dependent depolarization of the RMP, without affecting the number of action potentials (AP) fired upon application of depolarizing current pulses. We were also able to confirm the blockade that verapamil exerts on voltage-dependent potassium (*I*_Kv_) currents *I*_KDR_ and *I*_A_. Probably more important, we show for the first time that verapamil induces a robust inhibition of TREK channels activated by riluzole.

## Material and methods

Swiss CD1 mice were obtained from the Biomedical Research Center (CINBIO) of the University of Vigo. Mice were housed under 12 h light/dark cycle in a pathogen-free area, with food and water freely available. All experiments were approved by the Spanish Research Council and the University of Vigo Scientific Committee, under Spanish and European directives for the protection of experimental animals (RD 05/03/2013; EU 06/03/2010).

### Culture of mSCG neurons

The culture of mSCG neurons was performed as described previously (Romero et al., 2004). Mice (30–60 days of age) were terminally anesthetized with CO_2_ and immediately decapitated. The SCG were removed in cold Leibowitz medium (L-15) under a binocular microscope and, once cleaned, the ganglia were incubated in collagenase (2.5 mg/ml in HBSS) for 15 min at 37°C. The ganglia were then incubated for 30 min in trypsin (1 mg/ml). Finally, the neurons were mechanically isolated, centrifuged, and plated in 35 mm Petri dishes previously treated with laminin (10 μg/ml in EBSS). Neurons were cultured for 1 day at 37°C and 5% CO_2_ in L-15 medium containing the following: 24 mM NaHCO_3_, 10% fetal calf serum, 2 mM l-glutamine, 38 mM D-glucose, 100 UI/ml penicillin, 100 μg/ml streptomycin and 50 ng/ml nerve growth factor.

### Perforated-patch whole-cell recordings

On the day of the experiment, cultured mSCG neurons were placed on an inverted microscope and continuously perfused by gravity (10 ml/min) with a standard solution at room temperature. Recordings were obtained using a HEKA (EPC 800) amplifier. Sampling frequency was 2 kHz (filtered at 0.5 kHz) for voltage-clamp and 10 kHz (filtered at 5 kHz) for current-clamp (Bridge mode) experiments. Patch pipette resistance varied from 4 to 6 MΩ. Data were digitized using a Digidata 1440A and analyzed offline using the software pClamp10 (Molecular Devices). Plotting and statistical analysis were performed with Origin (Pro) 8.5 (OriginLab Corporation, Northampton, MA, United States). Membrane conductance (*G*) was estimated in voltage-clamp experiments (holding potential = −30 mV) by the application of negative 15 mV brief voltage steps (50 ms) at 0.5 Hz. After measuring the current (*I*) obtained during these steps, *G* was calculated using the Ohm’s Law, where *G* = 1/*R* and *R* = 15 mV/*I*. Assuming that the Perforated whole-cell Patch-Clamp limits washout and drift problems to the maximum. We took 20 random points before and after treatment and compared the average conductances of each cell in the two conditions (control and drug-treated).

### Chemicals

The intracellular pipette solution contained (in mM) 90 K-acetate, 20 KCl, 3 MgCl_2_, 1 CaCl_2_, 3 EGTA, and 40 HEPES (pH adjusted to 7.2 with NaOH), and the standard extracellular solution (standard solution) contained (in mM) 140 NaCl, 3 KCl, 1 MgCl_2_, 2 CaCl_2_, 10 D-glucose and 10 HEPES (pH adjusted to 7,2 with Tris (Tris (hydroxymethyl)-amino methane)). All solutions were kept between 290 and 300 mOsm.When specified, tetraethylammonium chloride (TEA, 15 mM), 4-aminopyridine (4-AP, 2 mM), cesium chloride (CsCl, 1 mM), cadmium chloride (CdCl_2_, 100 μM) and TTX (0.5 μM), were added to the extracellular solution in order to block voltage-dependent potassium, cationic, calcium and sodium currents. All these chemicals were purchased from Sigma-Aldrich. This combination of drugs constituted the standard cocktail solution (referred to as Cocktail A). In some experiments, this cocktail was supplemented with apamin (200 nM), paxilline (1 μM), 4-aminopyridine (4-AP, 2 mM) and clemizole (10 μM) (referred to as Cocktail B) in order to block, respectively, calcium dependent potassium channels (SK and BK), TRP channels and A-Type potassium channels. All these chemicals were purchased from Tocris Bioscience. Verapamil (Sigma-Aldrich) was dissolved in distilled water (10 nM stock solution), and maintained in darkness and cold. Riluzole (Tocris Bioscience) was made up in DMSO at a stock concentration of 10 mM. Final DMSO concentration ranged 0.1–0.2%, which has been tested not to affect cell physiology.

### Statistical analysis

Data are represented as the mean ± SEM and statistical differences were assessed using a Paired Sample *t-Test* or a Two Sample *t-Test*. For the comparison of more than two groups the One-way ANOVA test has been applied. The differences among groups were considered significant when *p < 0.05, **p < 0.02 or ***p < 0.01. For the calculation of the TAU (63% of decay) value of the A-current decay, a mono-exponential curve fit based on the following equation for one term was applied using Clampfit 10.7 (Molecular Devices, United Kingdom):

$$f\left(t\right)=\overset{N}{\underset{i=1}{\sum }}A_{j}e_{+C}^{-t∕\tau_{i}}$$

Dose-response curves were fitted using the Hill equation:

$$y=START+\left(END-START\right)\frac{x^{N}}{K^{N}+x^{N}}$$

where *K* corresponds to the *EC*_50_ and *N* is the Hill coefficient.

## Results

### Verapamil depolarizes the membrane potential at rest without affecting the firing rate of mSCG neurons.

**FIGURE 1:**
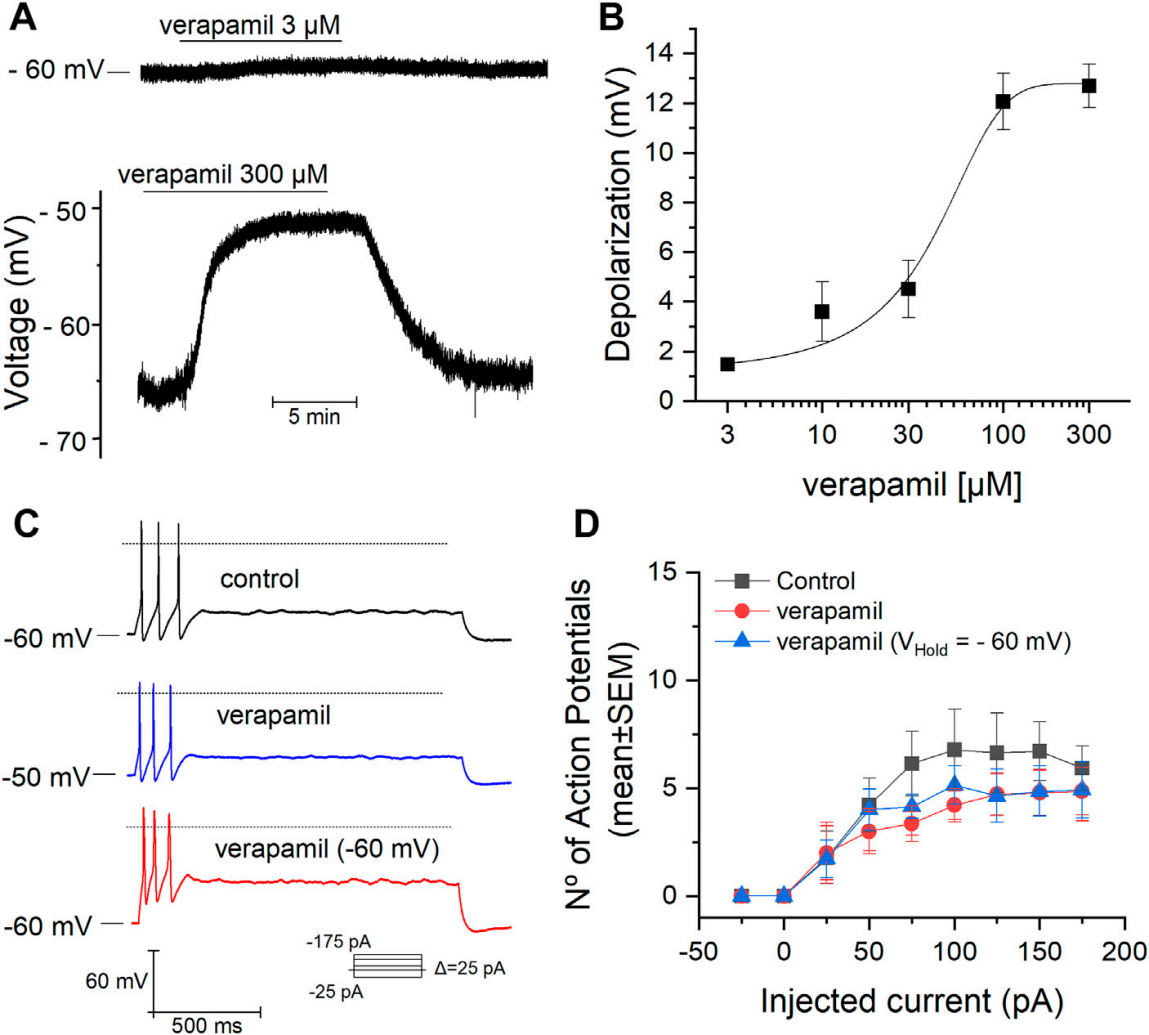
Effect of verapamil on the RMP and firing properties. **(A)** Membrane potential measured in CC conditions (gap-free mode). The addition of verapamil causes a clear depolarization when applied at 300 µM (bottom). **(B)** Dose-response curve for the verapamil-induced depolarization of the RMP. The depolarization induced by verapamil 3, 10, 30, 100 and 300 μM was of 1.5 ± 0.2 mV (*n* = 10), 3.5 ± 1 mV (*n* = 6), 4.5 ± 1 mV (*n* = 7), 12 ± 1 mV (*n* = 8) and 13 ± 1 mV (*n* = 8) respectively. **(C,D)** Number of action potentials (mean ± SEM) elicited in response to a protocol of increasing depolarizing currents pulses in the absence (black) and presence of verapamil (blue) and verapamil with the RMP hold at -60 mV (red). The dotted line represents the level at 0 mv.

We studied the effect of verapamil on the RMP of mSCG neurons, these neurons showed a mean membrane potential of -64 ± 3 mV (*n* = 39) at rest. The acute application of different concentrations of verapamil (from 3 to 300 µM) induced a dose-dependent depolarization of the membrane potential (Figures 1A,B) a maximal depolarization of 13 mV was obtained at 300 µM. Data points were fitted using the Hill equation, with an extrapolated half-maximal (*EC*_50_) concentration of 50.19 µM and a Hill coefficient of 0.82. This Hill coefficient is consistent with a single binding site (Monod et al., 1965; Weiss, 1997). Regardless of the clear depolarization induced by verapamil on the RMP, the addition of 50 µM verapamil did not affect the number of action potentials of mSCG neurons (*n* = 14) in response to depolarizing current pulses (Figure 1C). Because verapamil induces a depolarization of the RPM, after verapamil was added we set the RMP at −60 mV (V_Hold_ = −60 mV) to confirm that the lack of effect of verapamil on the firing pattern was independent on the membrane potential before the current injections (Table 1; Figure 1D).

**TABLE 1:** Number of action potentials elicited in response to depolarizing current steps in the presence and absence of verapamil. The values are expressed as mean ± SEM. Average number of AP in control condition, after the addition of verapamil, and in the presence of verapamil plus fixation of the RMP at -60 mV are indicated in columns. The *p* values were obtained with a One-way repeated measures ANOVA test followed by a Bonferroni post-hoc test, the average AP obtained in the three conditions was compared within each intensity.

Injected current	Avg ±SEM (Control)	Avg ±SEM verapamil	Avg ±SEM (VP + V_Hold_ = -60 mV)	ANOVA *p* value (*n* = 14)
25	1.78 ± 1.21	2 ± 1.24	1.71 ± 0.87	0.12
50	4.21 ± 1.24	3 ± 0.9	4 ± 0.95	0.57
75	6.14 ± 1.49	3.35 ± 0.54	4.14 ± 0.56	0.52
100	6.78 ± 1.88	4.21 ± 0.77	5.14 ± 0.88	0.32
125	6.64 ± 1.84	4.71 ± 0.96	4.64 ± 1.24	0.41
150	6.71 ± 1.35	4.78 ± 1.1	4.85 ± 1.16	0.11
175	5.92 ± 1.04	4.85 ± 1.1	4.92 ± 1.31	0.14

### Potassium delayed-rectifier and A-type, but not M-Type, potassium currents, are inhibited by verapamil

**FIGURE 2:**
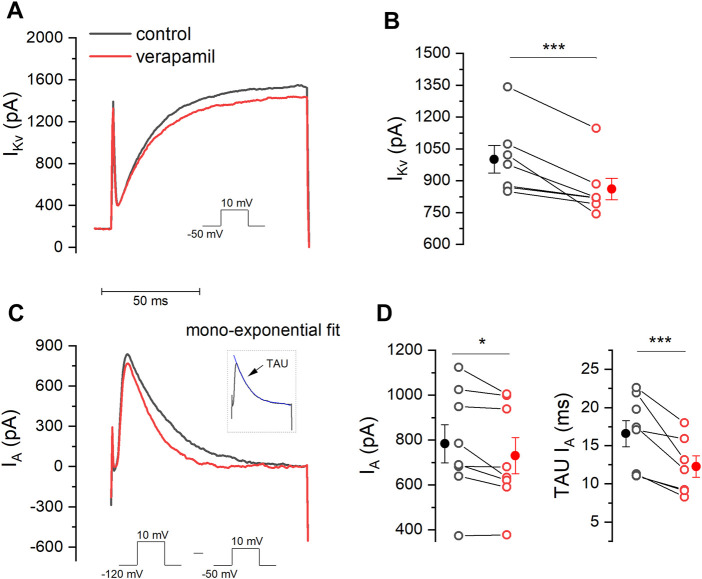
Effect of verapamil on *I*_Kv_ and *I*_A._ **(A)** A step protocol (−50 mV–10 mV) was elicited in order to activate *I*_Kv_ in the absence and presence of verapamil. *I*_Kv_ was measure at the end of this step. **(B)** Verapamil causes a significant reduction in the amplitude of the *I*_Kv_ current (*n* = 7, *p* = 0.005, paired sample *t*-Test). **(C)** To measure *I*_A_ we subtracted the current elicited by a step protocol from −120 mV to 10 mV from that evoked by the protocol used in. **(A)**. Inset illustrates how TAU (blue) was estimated. **(D)** 50 μM verapamil reduces peak current (*n* = 7, *p* = 0.034, paired sample *t*-Test) and accelerates the rate of decay, represented by the TAU (*n* = 7, *p* = 0.009, paired sample *t*-Test). Filled dots and error bars represent the mean ± SEM.

To evaluate the effect of verapamil on the IKv current, a (100 ms) voltage-clamp protocol was applied using a voltage step from -50 to + 10 mV, in the presence of TTX 0.5 μM and CdCl2 100 μM, and IKv was measured at the end of this step (Figure 2A). The application of verapamil (50 μM) induced a significant decrease of IKv from 1001.14 ± 65.05 pA in control conditions to 860.63 ± 50.27 pA in the presence of the drug, reducing the IKv current by 13.5 ± 2.92 % (Figure 2B, *n* = 7).

The experimental protocol for the recording of the A-type current is shown in Figure 2C. Two different currents are activated (*I*_Kv_ + *I*_A_) simultaneously when a voltage step from -120 mV to +10 mV is given, however a step from -50 mV to 10 mV will only activate *I*_Kv_ (Lamas 1997). It is therefore possible to obtain the A current in isolation by subtracting the second recording from the first one, thus allowing to fit the A current inactivation and to measure the inactivation time constant. *I*_A_ was measured at the maximum of the peak (Figure 2C and inset). Using this strategy, we found that verapamil induced a decrease of A current, showing 783.46 ± 85.49 pA in control conditions versus 730.60 ± 80.0 pA in the presence of verapamil. Also, we found that verapamil decrease of the *I*_A_ inactivation time constant. In control conditions, *I*_A_ TAU was 16.06 ± 1.9 ms, which was significantly reduced to 12.21 ± 1.4 ms after the application of 50 µM verapamil, reducing the inactivating TAU value by 22.9 ± 4.1% (Figure 2D).

**FIGURE 3:**
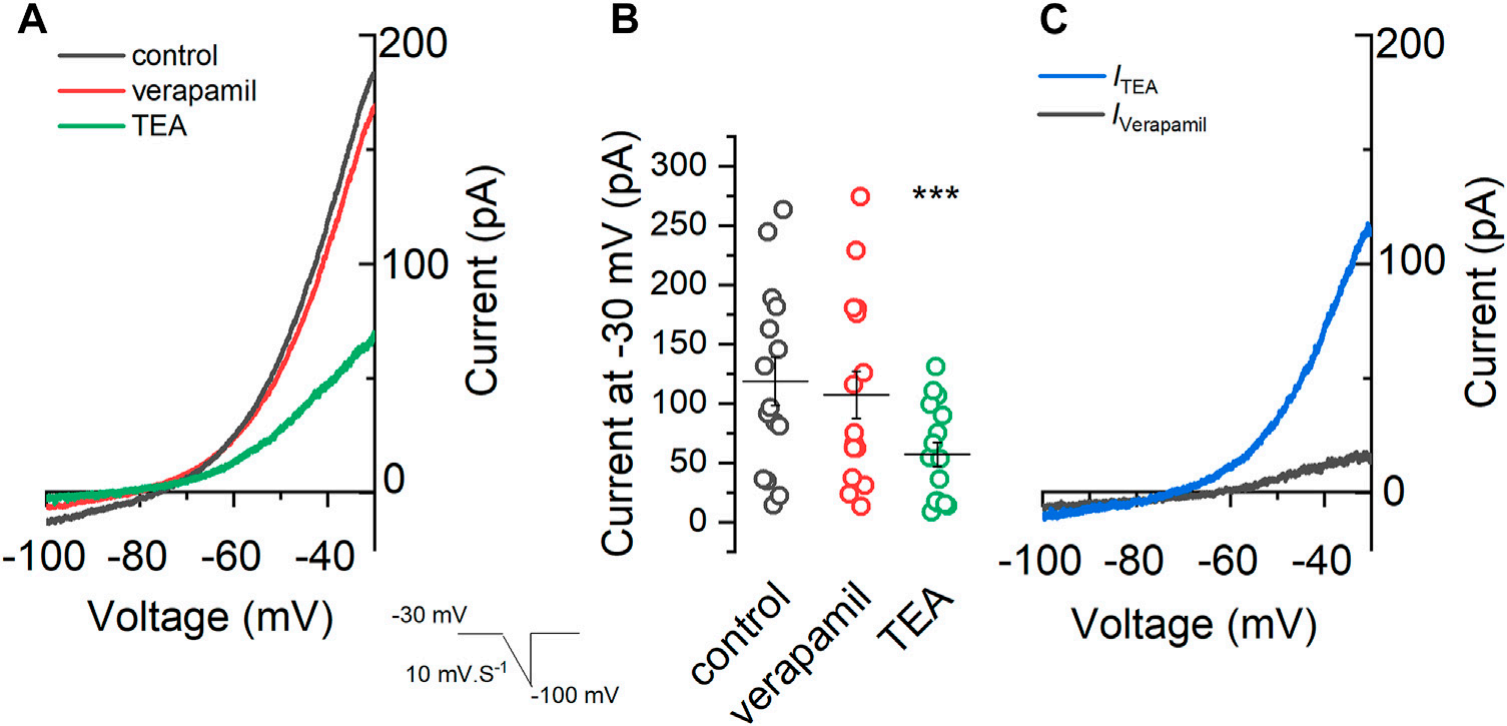
Effect of verapamil on the M-current. **(A)** A voltage-ramp from −30 mV to −100 mV (7 s) was applied in the presence of 0.5 μM TTX, 100 μM CdCl_2_ and 1 mM CsCl (control condition, black) in order to activate the M-current. After that 50 μM verapamil (red) and 15 mM TEA (green) were sequentially added . **(B)** Summary of the current values at −30 mV under different conditions. We found a clear difference among the currents observed in the presence of TEA, in control conditions and in the presence of verapamil (*n* = 14, One-way repeated measures ANOVA, Bonferroni test, *p* = 0.00004 and *p* = 0.00003, respectively), while no difference was found between current in control conditions versus verapamil (*p* = 1). **(C)** The TEA-sensitive (*I*_TEA_) current (blue) is obtained by subtracting the control (A, black) current from the current in the presence of TEA (A, green). Showing a clear rectification at values below −60 mV and almost no current at value below −60 mv, as expected for the M-current. Contrarily, the verapamil-sensitive current (*I*_Verapamil_), obtained by subtracting the control current and the current in the presence of verapamil, showed a linear leak-like kinetic.

Besides *I*_Kv_ and *I*_A_, we also wanted to evaluate whether verapamil is an inhibitor of the M-current. The M-current, known to be blocked by TEA (Hadley et al., 2003), was activated using voltage-ramps ranging from -30 to -100 mV (7 s) in the presence of TTX 0.5 μM, CdCl_2_ 100 μM and CsCl 1 mM. As seen in Figure 3A, the application of verapamil did not significantly change the magnitude of the current at any level of the ramp. In control conditions, the current at -30 mV was 118.39 ± 20.29 pA (black trace), and when verapamil was added, the current was 107.23 ± 19.74 pA (red trace). On the contrary, when TEA 15 mM was applied (green trace) a clear inhibition of the outward current was observed, and the current elicited at -30 mV (56.91 ± 10.26 pA) was significantly smaller than that observed in control and in verapamil conditions (Figure 3B). Next, we evaluated the kinetics of the currents sensitive to both drugs TEA and verapamil. For this, we subtracted from the control the current in the presence of TEA and verapamil (*I*_TEA_ and *I*_Verapamil_ respectively). Both currents show a different kinetics, while the *I*_TEA_ (blue) is clearly open at values above -60 mV, the *I*_Verapamil_ (grey) has a linear kinetic, much like a leakage (Miranda et al., 2013) current that is slightly open at all voltages from -100 to -30 mV (Figure 3C).

### The verapamil-induced membrane depolarization is associated with a current sensitive to the TREK channel blocker fluoxetine

**FIGURE 4:**
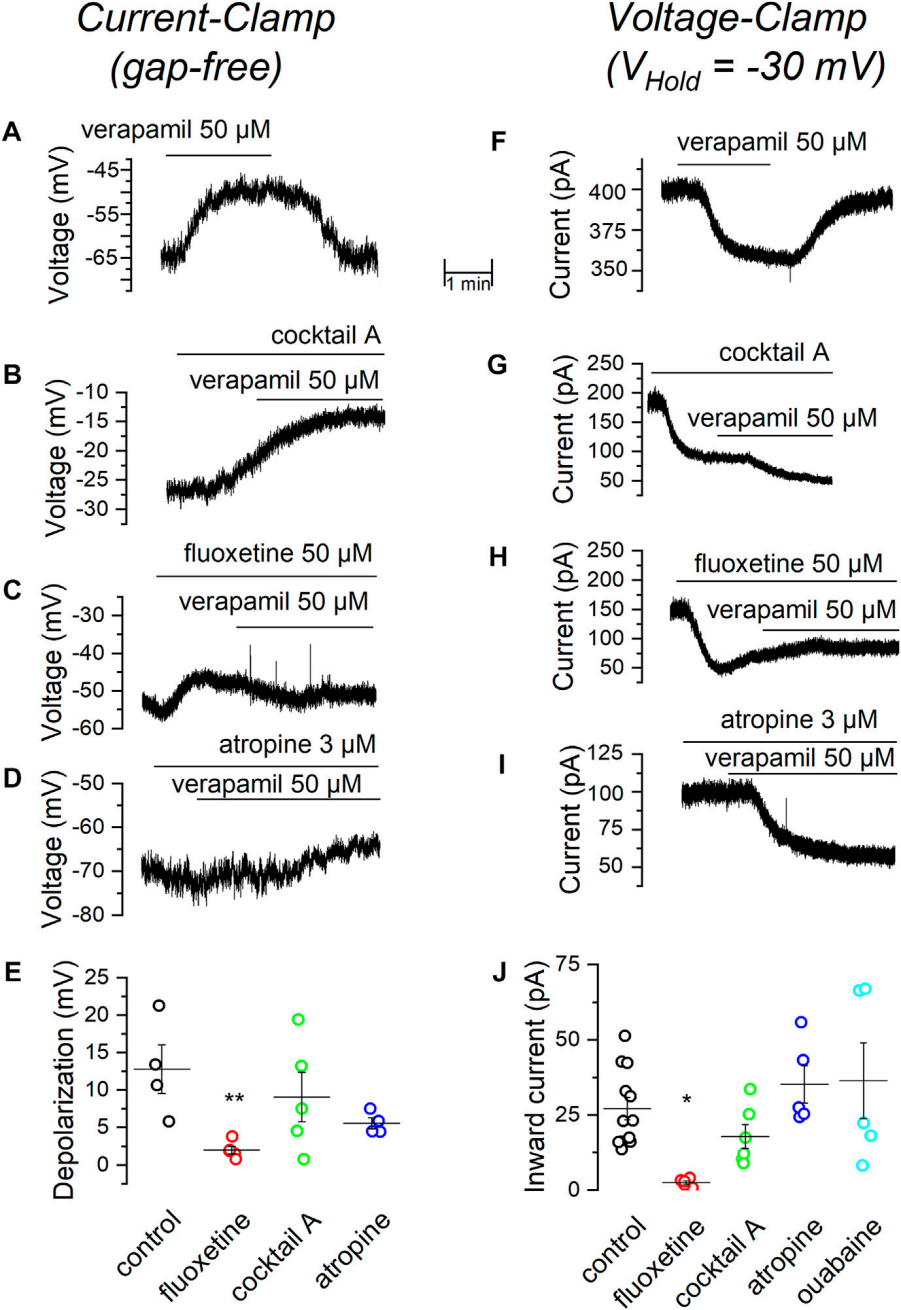
Effect of verapamil in the presence of different blockers. **(A)** Depolarization induced by 50 µM verapamil in current-clamp (gap free) conditions. In the presence of the standard cocktail of blockers. **(B)**, fluoxetine **(C)** and atropine **(D)**. A summary of results comparing the change in membrane potential elicited by 50 µM verapamil under different conditions is shown in. **(E,F)** In voltage-clamp conditions (*V*_Hold_ = −30 mV), current induced by 50 µM verapamil alone. **(F)**, in the presence of the standard cocktail of blockers **(G)**, fluoxetine **(H)** and atropine **(I)**. A summary of results comparing the inward current induced by 50 µM verapamil under different conditions is shown in **(J)**. Values are summarized in Table 2, **p = 0.01, *p = 0.04.

The voltage-ramps shown in Figure 3C suggest that verapamil might be inhibiting a voltage-independent potassium leak current and we know that SCG neurons do express a good amount of leak TREK2 channels (Cadaveira-Mosquera et al., 2012). To check this hypothesis, we tested the effect of verapamil in the presence of several channel blockers and antagonists using the current-clamp bridge-mode technique (Table 2). Initially, we tested whether a combination of drugs, that we know does not affect the TREK channels (Cadaveira-Mosquera et al., 2011), could prevent the depolarization normally induced by verapamil. This mixture (called Cocktail A) contained blockers of sodium (TTX), potassium (TEA), calcium (cadmium) and cationic h (cesium) currents. We found that the depolarization evoked by verapamil in the presence of the cocktail A (Figure 4B; Table 2) is similar to that found in control conditions (Figures 4A,E), supporting the hypothesis that such effect is not due to voltage-dependent ion channels.

**TABLE 2:** Verapamil depolarizing effect in both current- and voltage-clamp. Significance was determined using a One-way ANOVA test followed by a Bonferroni post-hoc test (**p = 0.01 and *p = 0.04, respectively).

Mode	Drug	RMP ±SEM	n	Mode	Drug	Inward current ±SEM	n
Current-Clamp (gap-free)	Control	12.7 ± 3.2 mV	4	Voltage-Clamp (V_Hold_ = -30 mV)	Control	27.1 ± 3.7 pA	6
	Cocktail A	9.1 ± 3.3 mV	4		Cocktail A	17.87 ± 4 pA	6
	Fluoxetine	2 ± 0.5 mV**	5		Fluoxetine	2.5 ± 0.6 pA*	5
	Atropine	5.6 ± 0.7 mV	4		Atropine	35.2 ± 6.2 pA	5
					Ouabain	36.3 ± 12.6 pA	5

Because a major component of the currents stabilizing the resting membrane potential in mSCG neurons is driven by leak channels of the TREK subfamily (Cadaveira-Mosquera et al., 2011; Rivas-Ramírez et al., 2020), we decided to investigate whether these channels are responsible for the verapamil-induced membrane depolarization seen in our previous experiments. For this purpose, we applied verapamil in the presence of the TREK channel blocker fluoxetine (Kennard et al., 2005). The presence of 50 µM fluoxetine strongly prevented verapamil from inducing membrane depolarization (Figures 4C,E), suggesting again that the inhibition of TREK channels by verapamil could account for its depolarizing effect.

It has been reported that muscarinic agonists inhibit TREK currents in SCG neurons by reducing PIP_2_ (Lamas et al., 2012; Rivas-Ramirez et al., 2015). We wondered whether the effect of verapamil could be somehow related to the interaction of this drug with the muscarinic cascade. For this purpose, we added verapamil in the presence of atropine, a general muscarinic antagonist. Atropine failed to affect the membrane depolarization induced by verapamil (Figure 4D). In summary, only the presence of the TREK blocker fluoxetine was able to reduce the depolarization produced by the addition of verapamil (Figure 4E).

### Voltage-clamp experiments support the hypothesis of the implication of TREK channels on the depolarization produced by verapamil

As a next step, we focused into the current responsible for the depolarization induced by verapamil and therefore performed a series of experiments in the voltage-clamp configuration. With the membrane potential clamped at -30 mV (Cadaveira-Mosquera et al., 2011), the application of 50 µM verapamil induced an inward current (Figure 4F; Table 2) which, as in CC conditions, was insensitive to the application of the cocktail A (Figure 4G). This verapamil-evoked current was however completely suppressed in the presence of the TREK channel blocker fluoxetine (Figure 4H). As in CC mode, 3 µM atropine had no effect on the current-induced by verapamil (Figure 4I). To discard the possibility that the verapamil-induced inward current could be produced by the activation of the Na^+^/K^+^ pump, in an additional set of experiments we tested 500 µM ouabain (Lamas et al., 2002; Mahmmoud et al., 2014), which also failed to affect the verapamil-induced inward current. In agreement with the result obtained in current-clamp mode, in voltage-clamp mode only the presence of the TREK inhibitor was able to block the effect of verapamil (Figure 4J).

### Verapamil induces a dose-dependent inhibition of TREK currents

**FIGURE 5:**
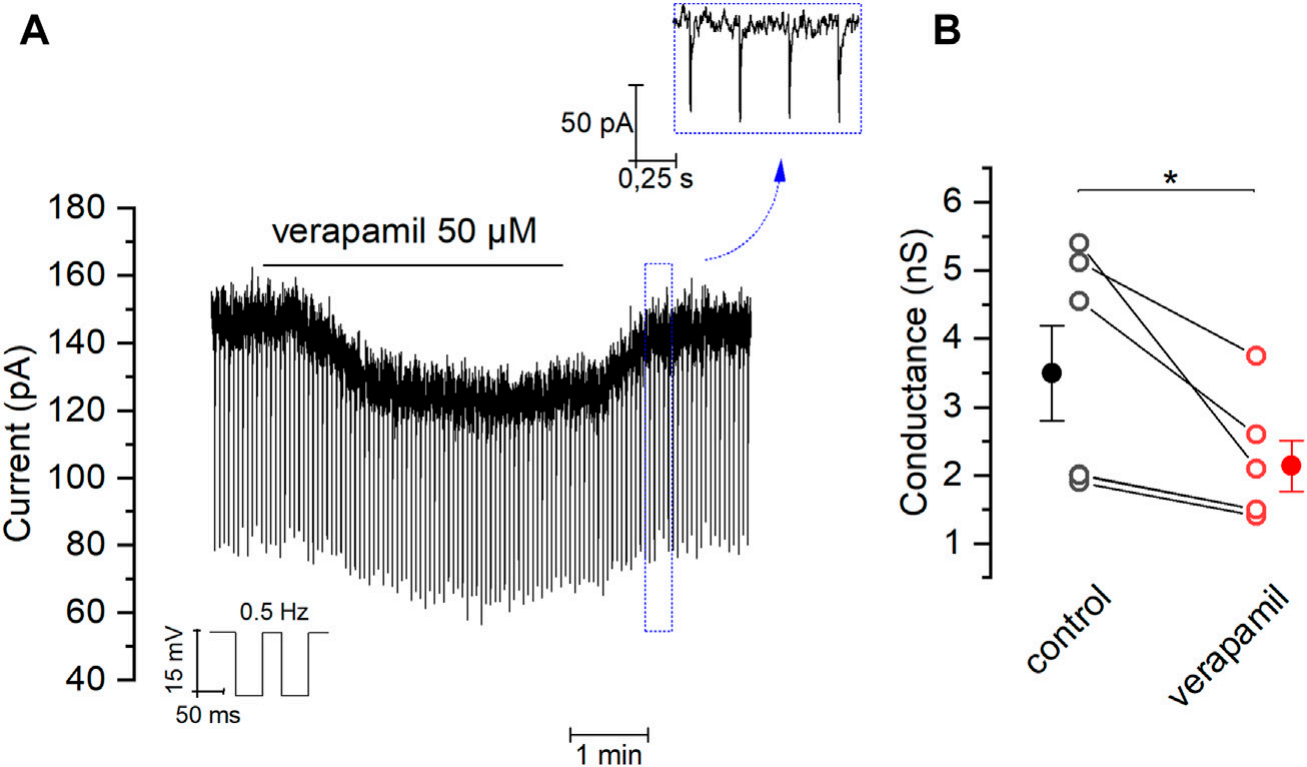
Effect of verapamil on the membrane conductance **(A)** Brief negative pulses (−15 mV at 0.5 Hz) were applied from a holding potential of −30 mV in order to measure the membrane conductance. We chose 20 random measurements of conductance (see material and methods) in absence and presence of verapamil, comparing the average for each cell **(B)** Effect of verapamil in conductance (*n* = 6, paired sample t-Test, *p = 0.03). Filled dot and error bars represents mean ± SEM.

Our previous experiments suggest that the inhibition of TREK channels could account for the depolarization induced by verapamil in mSCG neurons. If this were true, verapamil should increase membrane resistance. In order to confirm this hypothesis, we clamped the membrane potential at -30 mV and analyzed its conductance in the presence and absence of the drug (see Methods). Figure 5A shows how the application of verapamil reduces the size of the small currents evoked by the hyperpolarizing pulses used to measure membrane conductance from 3.49 ± 0.69 nS in control conditions to 2,14 ± 0.37 nS in the presence of 50 µM verapamil (Figure 5B). This indicates that the closure of outward currents, presumably through TREK channels, and not the activation of inward currents, is the responsible for the effect of verapamil in our cells.

**FIGURE 6:**
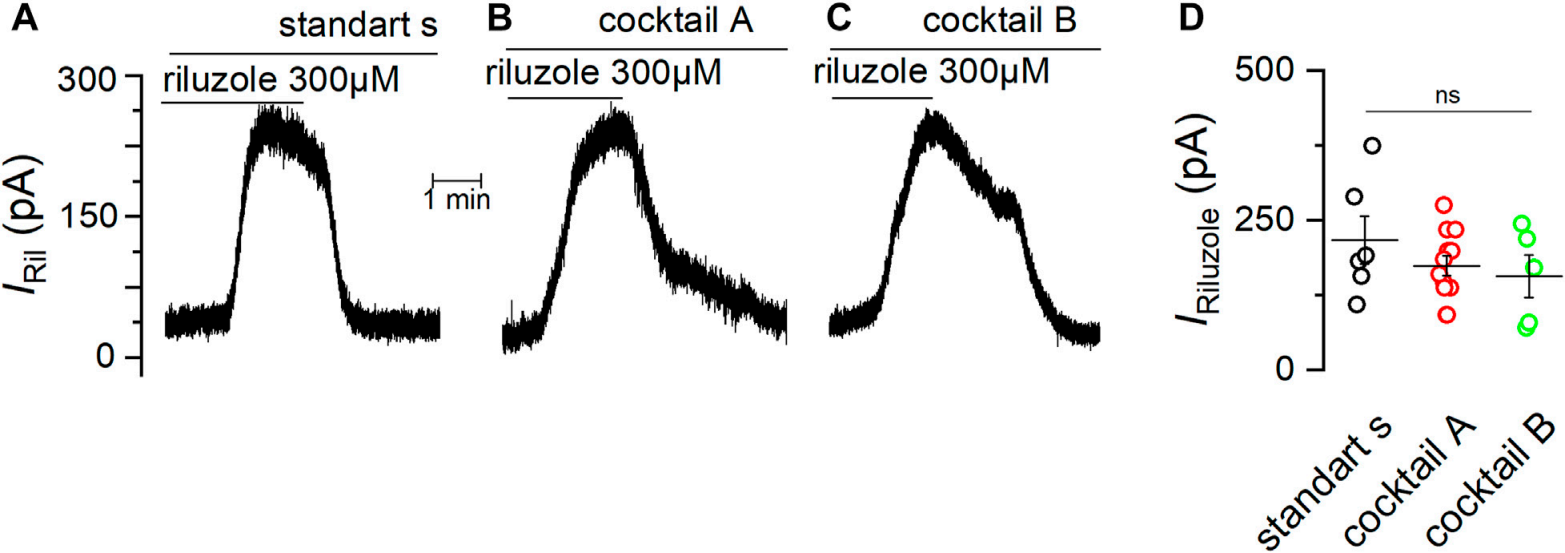
The current induced by riluzole is unaffected by different channel blockers. Experiments in VC mode with membrane potential fixed at −30 mV showing the outward current induced by 300 µM riluzole in standard solution **(A)**, in the presence of cocktail **(A,B)** and in the presence of the cocktail **(B,C)** are shown **(D)** Summary of the current induced by riluzole (*I*_RIL_) under different conditions. Significance was determined using a one-way ANOVA test (*p* = 0.37).

We previously showed that TREK-2 and TRESK are the most abundant members of K2P channels expressed in the mSCG (Cadaveira-Mosquera et al., 2012). Therefore, we decided to test if the effect of verapamil is specifically related to the activation of TREK-2 and not TRESK channels. For this purpose, we applied riluzole, which in the presence of cocktail A, induces the activation of TREK-2 channels (Duprat et al., 2000; Cadaveira-Mosquera et al., 2011), and at the same time inhibits TRESK channels (Fernández-Fernández et al., 2018). With the membrane clamped at -30 mV, the application of riluzole in standard solution evoked an outward current (*I*_RIL_) of 216.7 ± 39.7 pA (*n* = 6) (Figure 6A). As we expected, *I*_RIL_ was unaffected by the presence of cocktail A (see Methods) (173.8 ± 16.5 pA, *n* = 12) (Figure 6B) or in the presence of a more complete cocktail (cocktail B) aiming to additionally block calcium-dependent potassium channels (SK and BK) and TRP channels (156.5 ± 35.3 pA, *n* = 5) (Figure 6C). Taken together, these data confirm that, in mSCG cells, *I*_RIL_ is indeed driven by TREK-2 channels, not finding significant differences between the conditions (Figure 6D).

**FIGURE 7:**
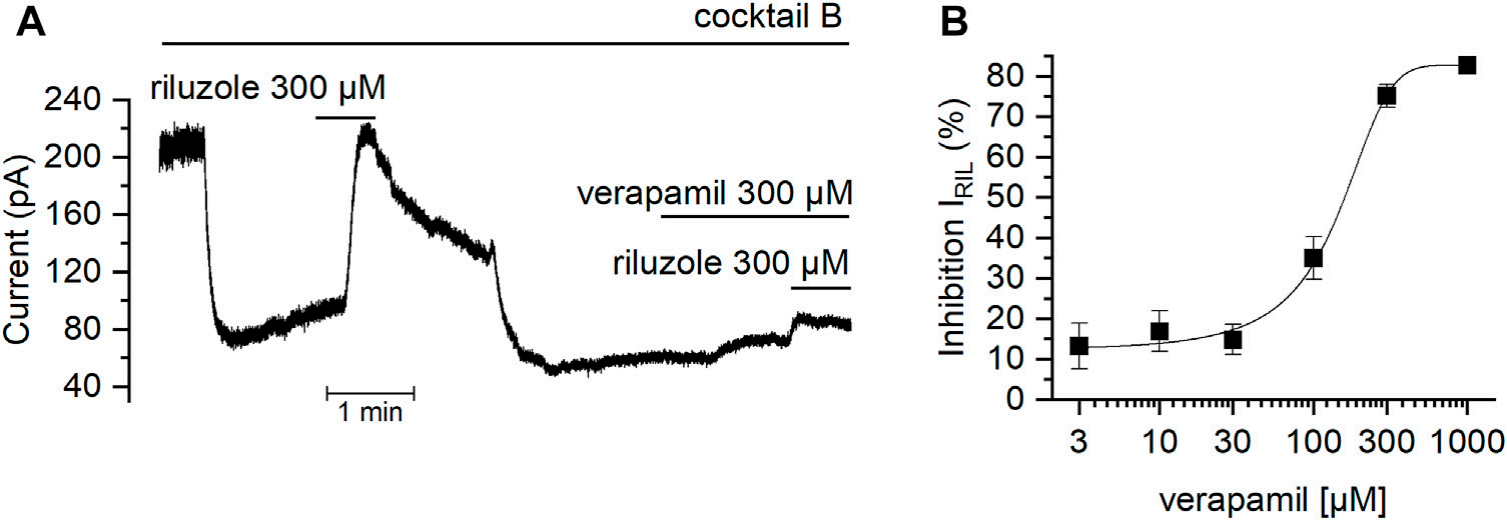
Effect of verapamil on the current activated by riluzole **(A)** With the membrane potential clamped at −30 mV in the presence of cocktail B (see Methods), the TREK activator riluzole was added in the absence and in the presence of verapamil. Note that when verapamil is present, the current activated by riluzole is strongly reduced. **(B)** Dose-dependent effect of verapamil over the current activated by riluzole.

After verifying that the *I*_RIL_ was driven by TREK channels in mSCG, we investigated whether the riluzole-activated current was modified by verapamil. Figure 7A shows how the presence of verapamil strongly reduced *I*_Ril_ obtained in cocktail B. The effect was dose-dependent (Figure 7B) and inhibition values reached by 1000, 300, 100, 30, 10 and 3 μM verapamil on *I*_RIL_ were: 82.7 ± 0.2% (*n* = 4), 75.15 ± 0.5% (*n* = 5), 35 ± 1% (*n* = 5), 14.8 ± 0.7% (*n* = 4), 16.9 ± 1% (*n* = 4) and 13.3 ± 1.6% (*n* = 6) respectively. Data points were fit using the Hill equation with an estimated *IC*_50_ of 96.09 μM and a Hill coefficient of 1.3. It has been suggested that the binding site of verapamil on other potassium channels located intracellularly (Cohen et al., 1987; Rauer and Grissmer, 1999) and that their adhesion causes a collapse of the pore (Baba et al., 2015). However, it is also described that verapamil might exert its blockade by interacting with the extracellular side of these channels (Keith et al., 1994). Our dose-responses curves of verapamil with both the membrane depolarization and *I*_RIL_ report a Hill coefficient that suggest that this effect happens through a similar mechanism which might require at least two interaction sites acting cooperatively in the channel (Monod et al., 1965; Weiss, 1997).

## Discussion

### Verapamil depolarizes the resting membrane potential of sympathetic neurons

In the present study, we aimed to investigate the specific effects that the antiarrhythmic verapamil exerts on different potassium conductances evoked in isolated neurons from the mSCG, which is an important regulator of the heart within the sympathetic branch of the autonomic nervous system (Kawashima, 2005). For the first time, we show that verapamil causes a dose-dependent depolarization of the resting membrane potential of mSCG neurons. In the intracardiac ganglion of the parasympathetic system, verapamil has been reported not to affect the RMP, yet it causes a decrease in the firing rate of isolated neurons from this ganglion (Hogg et al., 1999). The depolarization of the RMP seen in our experiments does not lead to an increase in the number of action potentials fired in response to depolarizing current steps. This apparent inconsistency might be explained by the different repertory of ion channels present in both types of neurons, which in this way could account for the overall opposite effect that verapamil exerts in both sympathetic and parasympathetic autonomic nervous systems (Hogg et al., 1999; Sun et al., 2000). An important issue to take into account and that could constitute a limitation to this conclusion is the fact that all the recorded cells showed a phasic firing pattern (Figures 1C,D). Although this pattern has been shown to be preponderant in rodent SCG cells (Malin and Nerbonne, 2000; Martinez-Pinna et al., 2018), the fact that SCG cells with a tonic firing pattern could see their frequency affected cannot be ignored. Indeed, in our study we report a significant yet discrete inhibition of the potassium delayed rectifier channel current (*I*_Kv_) by 50 µM verapamil as shown before both in heterologous and native systems (Chouabe et al., 1998; Baba et al., 2015; Diesch and Grissmer, 2017; Orvos et al., 2019). However, in the intracardiac ganglion, 10 µM verapamil is already enough to inhibit more than half of *I*_Kv_ currents, claimed by the authors to be the underlying mechanism causing a decrease in AP firing upon verapamil application (Hogg et al., 1999). Additionally, we demonstrate for the first time that, in contrast to the sinoatrial and atrioventricular nodes cardiac action potential where verapamil exerts an increase in its refractory period (Nademanee and Singh, 1988), in mSCG cells this drug produces a decrease of both peak and acceleration of the TAU of *I*_A_. In this respect, although we have not found an increase in firing rate, it is possible that this is due to the low concentration used or the counteracting effect caused by the inhibition of *I*_Kv_ currents. On the other hand, a lower strength in and a faster inactivation, in other words, a reduction in *I*_A_, could lead to a reduction in the firing frequency that could be evident in cells with a tonic firing pattern. In fact, as shown in Figure 2D, there is a reduction in the number of action potentials, although this reduction was not significant. In agreement with previous reports, we also show here that bath application of verapamil does not affect the M-current (Chouabe et al., 1998; Hogg et al., 1999).

### Verapamil inhibits TREK channels in the mouse superior cervical ganglion

Our results show that the depolarization caused by verapamil in the mSCG neurons is caused by the blockade of an outward current, associated with a decrease in the membrane conductance and therefore a closure of channels. We have tested a combination of channel blockers in order to block voltage-dependent potassium, cationic, calcium and sodium currents, but this had no effect on the depolarization or the inward current induced by verapamil. The inability of TEA to prevent the depolarizing effect of verapamil would emphasize our finding that the M-current is insensitive to the drug. In the same way, the presence of atropine both in CC mode and in VC mode, and ouabain in VC mode, would rule out the participation of metabotropic receptors and Na^+^/K^+^ pump respectively as mediators of the effect of verapamil.

We have previously demonstrated that under our experimental conditions, a major component of the outward current seen under resting conditions is driven by K2P channels of the TREK subfamily (Cadaveira-Mosquera et al., 2011). In fact, of all the blockers used here only the application of fluoxetine, an inhibitor of TREK channels (Kennard et al., 2005; Heurteaux et al., 2006), was able to fully abolish the effect of verapamil in both voltage- and current-clamp conditions. Altogether, these results support our thesis that verapamil blocks TREK channels in mSCG neurons. Our dose-responses curves of verapamil reported a Hill coefficient that suggest that this effect happens through at least two interaction sites acting cooperatively in the channel. The polysite pharmacology of TREK channels is well known (Pope and Minor, 2021). It has been shown how TREK channels have several binding sites for small molecules found on the extracellular side of the channel, comprising at least four binding sites for these molecules including the keystone inhibitor site, the K2P modulator pocket, the fenestration site, and the modulatory lipid site. Each one offering a different and very rich structural environment for the control of the channels by the active molecules (Dong et al., 2015; Lolicato et al., 2017; Schewe et al., 2019). Therefore, it is not surprising that, like fluoxetine, verapamil shows affinity for more than one binding site in the structure of TREK channels.

Blocking the TREK channels was further confirmed by the fact that the neuroprotective agent riluzole, an activator of TREK subfamily channels (Duprat et al., 2000), evokes an outward current that was largely inhibited by the application of verapamil in a dose-dependent manner. Importantly, this inhibition takes place even in the presence of our standard cocktail of drugs also supplemented with apamin, paxilline, 4-aminopyridine and clemizole. This indicates that calcium, potassium and sodium voltage-gated currents, the h current, calcium dependent potassium channels (SK and BK) and TRP channels, are all not mediating the inhibitory effect that verapamil exerts on the current activated by riluzole, strongly suggesting that indeed TREK channels are those inhibited by verapamil. Among them, TREK-2 is most likely the main channel blocked by verapamil under these conditions, as it has been shown to be the most abundant TREK channel present in mSCG neurons (Cadaveira-Mosquera et al., 2011; Cadaveira-Mosquera et al., 2012). Alternatively, very recently the presence of TREK-2/TRESK heterodimers has been described in trigeminal primary sensory neurons (Lengyel et al., 2020). Although we have no news saying that chimera is expressed in the SCG, it cannot be ruled out. Supporting our hypothesis, the inhibition produced by verapamil in the presence of fluoxetine would strongly indicate that the effect of the drug would be driven mainly by TREK-2.

### Functional implications

The sympathetic system exerts a positive control over the sinoatrial node and the cardiomyocytes. In particular, the SCG functions as a relay station, from where postganglionar neurons synapse with the cardiac tissue (Hernandez-Ochoa et al., 2009; Peng-Sheng Chen et al., 2014). From the SCG, motor pathways that reach the heart set the activity of the sinoatrial and atrioventricular nodes, and downstream, the cardiomyocytes (Pather et al., 2003; Hernandez-Ochoa et al., 2009; Klabunde, 2011). Therefore, any alteration of this system could be harmful for the normal functioning of the heart. In fact, the SCG has been implicated in various cardiovascular conditions (Kong et al., 2013; Na et al., 2014; Murakami et al., 2015; Cheng et al., 2018). In the present study, we have shown that verapamil causes a clear depolarization of mSCG neurons by blocking TREK-2 channels, an effect which would contribute to the increase of sympathetic activity. Additionally, another explanation could be given. Although the maximum depolarization (∼10 mV induced by 300 μM) produced by verapamil would place the RMP at a value (∼ to -55 mV) in which the voltage-gated sodium channels would still be available (Vandael et al., 2015), it could be that a slower repolarization induced by a greater inactivation of the *I*_A_ together with an eventual lower availability of voltage-dependent sodium channels leads to an inactivation of the sympathetic system which could act synergistically with the reduction of the activity of the L-type calcium channels, promoting greater sympathetic-parasympathetic dysregulation. Although during atrial fibrillation (AF) verapamil causes a reduction in the nodal rhythm (Stern et al., 1982), the increase in excitation of peripheral sympathetic neurons by verapamil, described in the present study, could also lead to various pathological situations such as AF (Nguyen et al., 2009), the dysregulation of the sympathetic-parasympathetic balance (Zhang et al., 2009; Vaseghi et al., 2014), exercise-induced tachycardia (Ozdemir et al., 2003) and dysfunctions of the sinus rhythm (Elvan et al., 1996). It should be taken into account that the therapeutic concentration of verapamil in an adult of normal complexion can be in the nM range (Megarbane et al., 2011), so the effects shown *in vitro* (μM range) would have to be weighed and analyzed in a broader context. Anyway, considering the above, the therapeutic use of verapamil should take into consideration possible collateral effects of this drug on peripheral sympathetic relay stations such as the SCG.

## Data Availability

The raw data supporting the conclusion of this article will be made available by the authors, without undue reservation.
